# Tracing *Mycobacterium ulcerans* along an alimentary chain in Côte d’Ivoire: A one health perspective

**DOI:** 10.1371/journal.pntd.0008228

**Published:** 2020-05-28

**Authors:** Nassim Hammoudi, Sylvestre Dizoe, Jamal Saad, Evans Ehouman, Bernard Davoust, Michel Drancourt, Amar Bouam

**Affiliations:** 1 IHU Méditerranée Infection, Marseille, France; 2 Aix-Marseille Univ., IRD, MEPHI, IHU Méditerranée Infection, Marseille, France; 3 Programme National de Lutte contre l’Ulcère de Buruli (PNLUB), Kongouanou Care Center, Yamoussoukro, Côte d’Ivoire; 4 Université Nangui Abrogoua, UFR Sciences de la Nature, Abidjan, Côte d’Ivoire; Swiss Tropical and Public Health Institute, SWITZERLAND

## Abstract

**Background:**

*Mycobacterium ulcerans* is an environmental mycobacterium responsible for an opportunistic, noncontagious tropical infection named Buruli ulcer that necrotizes the skin and the subcutaneous tissues. *M*. *ulcerans* is thought to penetrate through breached skin after contact with contaminated wetland environments, yet the exact biotopes where *M*. *ulcerans* occurs remain elusive, hence obscuring the epidemiological chain of transmission of this opportunistic pathogen.

**Methodology/Principal findings:**

Polymerase chain reaction investigations detected *M*. *ulcerans* in 39/46 (84.7%) rhizosphere specimens collected in 13 Buruli ulcer-endemic areas in Côte d’Ivoire and 3/20 (15%) specimens collected in a nonendemic area (*P* = 5.73.E^-7^); only 3/63 (4.7%) sediment specimens from sediment surrounding the rhizospheres were positive in endemic area (*P* = 6.51.E^-12^). High-throughput sequencing further detected three PCR-positive plants, *Croton hirtus*, *Corton kongensis* and *Oriza sativa* var. *japonica* (rice), in the rectal content of two *M*. *ulcerans*-positive wild *Thryonomys swinderianus* grasscutters that were hunted in Buruli ulcer-endemic areas, while no PCR-positive plants were detected in the rectal content of two negative control animals that were farmed in a nonendemic area.

**Conclusions/Significance:**

Our data suggest an alimentary chain of transmission of *M*. *ulcerans* from plants to *T*. *swinderianus* grasscutters and people that utilize *T*. *swinderianus* as bush meat in Buruli ulcer-endemic areas in Côte d’Ivoire. Guidance to adopt protective measures and avoid any direct contact with potentially contaminated rhizospheres and with grasscutter intestinal content when preparing the animals for cooking should be established for at-risk populations.

## Author summary

Buruli ulcer caused by inoculated *Mycobacterium ulcerans* is a tropical infection fibrosing subcutaneous tissues thus causing severe disabilities. The reservoir and the mode of transmission of *M*. *ulcerans* remain elusive. Here, using molecular approaches, we traced *M*. *ulcerans* along an alimentary chain comprising some plants and a small herbivore named *Thryonomys swinderianus* (grasscutters), in Côte d’Ivoire. Grasscutters are hunted animals, sold as bush meat. People in Buruli ulcer endemic regions spend much time in close contacts with this animal during hunting and subsequent evisceration with unprotected hands increasing their risk of contamination. Our findings demonstrate for the first time, the transmission of *M*. *ulcerans* through a food-chain and propose prophylactic measures against Buruli ulcer in Côte d’Ivoire.

## Introduction

*Mycobacterium ulcerans* is a fastidious, slow-growing, opportunistic pathogen responsible for Buruli ulcer, a noncontagious tropical infection involving extensive necrosis of the skin and subcutaneous tissues, which occurs after *M*. *ulcerans* has been introduced through breached skin [1, 2]. *M*. *ulcerans* mycobacteria synthesize macrolide toxins called mycolactones that are encoded by three genes, MLSA 1, MLSA 2, and MLSB, and carried by a 174-Kb plasmid [1, 3]. Mycolactones induce cell apoptosis and exhibit anti-inflammatory and analgesic effects and are collectively thought to be responsible for the clinical and pathological features of Buruli ulcer [4].

Buruli ulcer is a World Health Organization (WHO) notifiable, neglected disease [5] reported in at least 34 countries, including rural tropical countries in Africa and South America as well as Japan [6, 7] and Australia, where the disease was initially described [8]. Buruli ulcer affects people of all races and ages, with a higher prevalence among adult women than among adult men [9]. In recent years, 83.6% of the total cases have been reported from several Western and Central African countries, including Côte d'Ivoire, which is one the most affected countries in the world, Benin, Ghana, the Democratic Republic of Congo and Cameroon [5,10]. In these Buruli ulcer-endemic countries, *M*. *ulcerans* is detected in wetland environments, including aquatic insects [6]. Even though *M*. *ulcerans* has been isolated from an aquatic Hemiptera in Benin and from *Thryonomys swinderianus* grasscutter feces in the Ivory Coast [11, 12], the exact ecosystems where *M*. *ulcerans* occurs in Africa remain unknown.

Clinical symptoms similar to those observed in humans have also been reported in several mammal species, including koalas, possums and dogs in Australia [13, 14, 15] and *Mastomys* spp. mice in West African countries [16]. These studies suggest a potential epidemiological role for animals in contact with people in Buruli ulcer-affected areas [16, 17, 18]. In addition, these observations indicated that in Buruli ulcer-endemic areas, people and mammals were probably exposed to the same sources of infection. We noted that 10/12 mammal species reported to be infected by *M*. *ulcerans* were herbivores; eating any part of the plants, including roots for herbivores of interest *T*. *swinderianus* in Côte d’Ivoire [19], this observation suggested focusing our search for environmental *M*. *ulcerans* on plants [6, 19]. Accordingly, the detection of *M*. *ulcerans* DNA has been reported in terrestrial vegetation in Australia [20]. Moreover, the acknowledged susceptibility of *M*. *ulcerans* to sunlight suggested focusing our research on the rhizosphere rather than the aerial parts of plants [6, 20].

To gain further insights into this question, we searched for *M*. *ulcerans* in some rhizospheres in Buruli ulcer-endemic areas in Côte d’Ivoire, and we subsequently confirmed the presence of some of these *M*. *ulcerans*-positive plants in the rectal content of *M*. *ulcerans*-positive wild *T*. *swinderianus* grasscutters. We compared wild *T*. *swinderianus* grasscutters to farmed *T*. *swinderianus* grasscutters, which served as a negative control that received controlled food; asserting for the first time that *M*. *ulcerans* may be part of an alimentary chain starting in the rhizosphere. Populations may be exposed to *M*. *ulcerans* through contact with bush meat, resulting in a potentially efficient and simple measure to avoid Buruli ulcer.

## Methods

### Environment sampling

The practice of environmental sampling was performed in agreement with the Nagoya protocol signed by Côte d’Ivoire. None of the plant or animal species investigated here is an endangered species. Plants roots were collected in 13 different locations in a 100-km perimeter around Yamoussokro, a Buruli ulcer endemic region in Côte d’Ivoire. In this region, the districts of Sinfra, Daloa, and Oumé reported nearly half of the new cases of Buruli ulcer in 2018 as notified to WHO. Also, Plants roots were collected in a non-endemic region of Grand Bassam 50 km east Abidjan where no case of Buruli ulcer has been notified to the WHO in 2018 [21]. Random sampling of roots was done by collecting roots after removal of the surrounding earth by simple shaking, and then the roots were cut with a single-use scalpel before being put into separate sterile tubes containing homemade sterile Trans MU transport media [11]. 51 wild *T*. *swinderianus* grasscutters were hunted by local populations in Buruli ulcer-endemic regions in Côte d’Ivoire without contravening the regulations: articles 433 and 434 of section 2 of the Penal Code of the Republic of Côte d'Ivoire. Rectal swabs from 51 wild *T*. *swinderianus* grasscutters and 23 feces specimens collected from 23 *T*. *swinderianus* grasscutters from livestock farms, which were used as negative controls were put in separate sterile tubes containing homemade sterile Trans MU transport medium. The tubes were stored at 4°C until molecular analysis.

### DNA extraction protocols

The collected samples, including the roots, feces, sediments and negative controls prepared in the field (PBS collected and manipulated exactly as the field samples), were subjected to molecular identification. After the manual lysis of root plants in the homemade sterile Trans MU transport medium using sterile disposable pestles, 500 μL of each sample was transferred to a 1.5 ml Eppendorf tube. A volume of 200 μL of lysis buffer G2, 20 μL of proteinase K (Qiagen GmbH, Hilden, Germany) and a small amount of glass powder was added. The samples underwent three runs of fast prep (6.5 m/s) followed by incubation at 56°C for three hours and centrifugation for one minute at 1,100 g. A 200-μL volume of supernatant was transferred into a new tube, and total DNA extraction was performed on an EZ1 machine (Qiagen). The extracted DNA samples were stored at -20°C until use.

### Molecular detection of *M*. *ulcerans* DNA in environmental samples

A-5μL volume of total DNA was incorporated into real-time PCR that targeted the IS*2404* and IS*2606* insertion sequences and ketoreductase-B domain of the mycolactone polyketide synthase (KR-B) gene [22]. Total DNA extracted from *M*. *ulcerans* CU001 grown on Middlebrook 7H10 agar plates at 30°C for six weeks was used as a positive control. DNA was replaced by sterile water in the negative controls (one negative control tube every 8 tubes), with the same reaction mixture. To estimate the *M*. *ulcerans* inoculum in each sample by extrapolation of the Ct values, a calibration curve was generated from an *M*. *ulcerans* CU001 DNA sample calibrated at one Mcfarland equivalent to 10^6^ colony-forming units (CFU)/mL, followed by 10-fold cascade dilutions up to 10^−1^ CFU/mL.

### Molecular identification of plants

Total DNA extracted from root samples was subjected to molecular identification by targeting the ITS2 gene [23]. Briefly, 5 μL of each DNA sample was amplified by standard PCR, and the latter was performed using HotStar *Taq* polymerase according to the manufacturer's instructions (Qiagen) in a thermocycler (Applied Biosystem, Paris, France). Electrophoresis on a 1.5% agarose gel was performed to separate the SYBR safe-stained PCR products (Thermo Fisher, Bourgoin Jallieu, France), and the bands were visualized under an ultraviolet transilluminator. The PCR products were purified using the Millipore NucleoFast 96 PCR kit according to the manufacturer's recommendations (Macherey-Nagel, Düren, Germany) and then sequenced using a BigDye Terminator Cycle Sequencing Kit (Applied Biosystems) with an ABI automatic sequencer (Applied Biosystems). The generated sequences were assembled using chromas Pro 1.7 software (Technelysium Pty Ltd., Tewantin, Australia) and compared to the NCBI database (http://blast.ncbi.nlm.nih.gov/Blastcgi) for species identification.

### Molecular identification of plant food in the feces of *T*. *swinderianus*

Two PCR-positive specimens and two negative controls were investigated for plant content by high-throughput sequencing of the PCR-amplified ITS2 sequence.

Similarly, DNA samples extracted from *T*. *swinderianus* feces were subjected to PCR amplification of the ITS2 intergenic sequence to identify the plant species on which *T*. *swinderianus* were feeding. The amplified DNA product was quantified by a Qubit assay with a high-sensitivity kit (Life Technologies, Carlsbad, CA, USA), and 0.2 μg/μL of DNA was sequenced with the Illumina MiSeq platform (Illumina Inc., San Diego, CA, USA). The DNA was fragmented and amplified by limited PCR (12 cycles), and dual-index barcodes and sequencing adapters were added. After purification with AMPure XP beads (Beckman Coulter Inc., Fullerton, CA, USA), the libraries were normalized and pooled for sequencing on the MiSeq platform. Paired-end sequencing and automated cluster generation with dual indexed 2× 251-bp reads were performed. A total of 14.5 Gb of information was obtained from a 1,168 k/mm2 cluster density, with a cluster quality control filter of 69.1%. Subsequently, the reads of each sample were assembled using SPAdes software [24]. Then, the highest number of contigs obtained from each sample was identified individually via a BLASTn-specific script against the NCBI database for nearly 5 hours. Finally, a simple-format Excel output containing the related species of each contig was generated. All positive reads identified in feces from wild *T*. *swinderianus* grasscutters were mapped with the *M*. *ulcerans* sequence (NC_008611.1) using CLC genomics7 (https://www.qiagenbioinformatics.com/products/).

### Statistical analysis

The statistical analysis of the results was carried out on the Biostat TGV website. Fisher’s exact test was used to calculate the P values for the detection of *M*. *ulcerans* DNA in root and sediment samples and the identification of the roots specifically associated with the presence of *M*. *ulcerans* in the feces of the positive animals.

## Results

### Plant identification and *M*. *ulcerans* detection in the rhizosphere

A total of 66 plant root specimens harvested in different regions of Côte d’Ivoire were identified. This investigation yielded a total of 32 different plant species from 14 geographic areas of collection. Twenty-eight plant species were found exclusively in Buruli ulcer-endemic regions, four were found exclusively in nonendemic regions and two were found in both endemic and nonendemic regions (S1 Table). While negative controls prepared in the field (PBS collected and manipulated exactly as the field samples) did not exhibit *M*. *ulcerans* DNA, real-time PCR detection of *M*. *ulcerans* (IS*2404*-IS*2606*-KR-B) in these plants showed that 39/46 (84.7%) specimens collected in Buruli ulcer-endemic areas were positive, and only 3/20 (15%) specimens collected in non-endemic regions were positive (*P* = 5.73.E^-7^) (Table 1). More precisely, 20/28 (71.4%) plant species collected in the Buruli ulcer-endemic region were carrying *M*. *ulcerans* (Table 1; S2 Table; S1 Fig).

**Table 1:** *IS2404*, *IS2606* and *KR-B* PCR results of root and sediment samples from Buruli ulcer endemic and non-endemic areas in Côte d’Ivoire.

*** ***	*M*. *ulcerans* PCR-positive from 63 sediment samples	*M*. *ulcerans* PCR-positive from 46 root plant samples from BU endemic areas	*M*. *ulcerans* PCR-positive from 20 root plant samples from non-endemic area s
***IS2404***	17	2	0
***IS2606***	0	0	0
***KR-B***	0	1	1
***IS2404-IS2606***	4	2	1
***IS2404-KR-B***	7	5	0
***IS2606-KR-B***	0	0	0
***IS2404-IS2606-KR-B***	3	39	3
**P-value (endemic/non-endemic areas)**	5.73. E-7		
**P-value (roots/ sedimens)**	6.51. E-12		

### Detecting *M*. *ulcerans* in the sediments

We observed that 3/63 (4.7%) sediment specimens were positive for *M*. *ulcerans* DNA by real-time PCR. The prevalence of *M*. *ulcerans* DNA in roots was significantly higher than the prevalence in surrounding sediments (*P* = 6.51.E^-12^) (Table 1).

### Detecting plants in *T*. *swinderianus* grasscutters

We identified 114 and 43 different plant species in wild grasscutters and farmed grasscutters, including 14 plant species that were identified in both wild and farmed grasscutters (S3 Table). The food mixture (hen eggs, soja and *Zea mays*) was negative for *M*. *ulcerans*, and Z. *mays* was detected in the feces of farmed grasscutters. Among 100 plants specifically associated with wild grasscutters, three plant species, *C*. *hirtus*, *C*. *kongensis* and rice, were positive for *M*. *ulcerans* during the rhizosphere investigations (S4 Table). More precisely, these three different plant species were significantly detected in *M*. *ulcerans-*positive grasscutters but not in the two-negative control grasscutters (S5 Table).

## Discussion

Three plant species, namely, *Croton hirtus*, *Croton kongensis* and *Oryza sativa* var. *japonica* (rice) positive for *M*. *ulcerans* have been collected in the same area where *Echinochloa crus-galli*, *Solanum lycopersicum*, *Hexachlamys emerichii* and *Castanopsis hystrix* were negative for *M*. *ulcerans*. These results, which were in agreement with some previous reports [25, 26], indicated some degree of specificity for the presence of *M*. *ulcerans* in the rhizosphere, suggesting that plants may be used as proxy indicators for *M*. *ulcerans* and Buruli ulcer, as reported previously in Ghana [25]. Accordingly, the presence of *C*. *hirtus* was previously reported in Buruli ulcer areas in Côte d’Ivoire [27] and recorded as an invasive plant associated with disturbed conditions [28]. Thus, *C*. *hirtus* may be a good indicator of disturbed environments associated with the presence of *M*. *ulcerans*.

We further investigated sediment specimens collected around the plant roots to specify the association between *M*. *ulcerans* and plant roots. Although limits in the specificity for detecting *M*. *ulcerans* DNA of the molecular detection method we used, have been previously reported [20], these limits may not prevent from comparing results from one sample to others. Accordingly, data showed that the prevalence of *M*. *ulcerans* DNA in roots was significantly higher than the prevalence in surrounding sediments. Accordingly, all previous studies indicated an absence of detection or the detection of low amounts of *M*. *ulcerans* DNA in soil in the Ivory Coast and Benin [29]. Additionally, these field observations agreed with our previous observation regarding the rapid death of *M*. *ulcerans* in experimentally inoculated soil [30]. Moreover, *M*. *ulcerans* is not able to survive in free-living amoebas; thus, amoebas cannot sustain the pathogen in soil [31]. However, in Australia, *M*. *ulcerans* DNA was detected in terrestrial vegetation [20], whereas in Buruli ulcer-endemic areas of Côte d’Ivoire, *M*. *ulcerans* was shown to be associated with aquatic plants [6, 32]. These previously published observations, along with new data reported here, suggest that in some Buruli ulcer-endemic regions, *M*. *ulcerans* exhibit a specific association with certain rhizospheres.

In the rhizospheres, *M*. *ulcerans* may live in complex microbial communities comprising, among others, some mycobacteria [33], fungi and algae [34]. Three groups of organisms have been previously suggested by the biochemical profiling of *M*. *ulcerans* [35]. Further studies may aim to establish the microbial repertoire of rhizospheres where *M*. *ulcerans* thrives to decrypt the interplay between *M*. *ulcerans* and its neighbors.

Three different plant species were detected in *M*. *ulcerans-*positive grasscutters but not in the two-negative control grasscutters. This association suggested that in Buruli ulcer-endemic regions in Côte d’Ivoire, herbivore grasscutters contaminate their intestinal tract after the ingestion of contaminated plant roots with *M*. *ulcerans*. The transmission of *M*. *ulcerans* from animals to people has been previously suggested by Fyfe and collaborators [20]. Therefore, the current report of *M*. *ulcerans* in *T*. *swinderianus* grasscutters is meaningful to the epidemiology of Buruli ulcer, as rural populations in endemic areas are in close contact with *T*. *swinderianus* grasscutters. *T*. *swinderianus* grasscutters are a nuisance species of rice; therefore, they are trapped and consumed as bush meat [36]. The preparation of *T*. *swinderianus* grasscutters consists of opening the abdomen and removing the viscera, including the intestines, prior to cooking. This procedure is usually performed with unprotected hands (authors’ personal observations). It is therefore possible that direct contact with *T*. *swinderianus* grasscutters and their viscera is one way to contaminate the skin with *M*. *ulcerans*; resulting in transcutaneous inoculation through any skin trauma.

This exploratory field investigation warrant further additional studies in order to strengthen the preliminary data here reported, overpassing potential limitations of the present study, multiplying the negative controls, using complementary methods of investigations such as whole genome sequencing and culture, investigating similar situations in other Buruli ulcer countries. The data reported in this study offer a One Health vision of Buruli ulcer, as the transmission cycle involves rhizospheres, herbivores and people in Buruli ulcer-endemic regions in Côte d’Ivoire. This study offers one possible scenario for the epidemiology of Buruli ulcer in this country, although this scenario is not exclusive and there may be other introduction scenarios for *M*. *ulcerans* complex mycobacteria in rural populations in Côte d’Ivoire. However, these results suggest an opportunity to promote an apparently simple prevention measure for populations in close contact with *T*. *swinderianus* grasscutters; avoiding direct contacts with roots and hunted animals by wearing any type of gloves to catch, handle and prepare plants and grasscutters as bush meat can potentially prevent Buruli ulcer infection.

## Supporting information

S1 Table Molecular identification of plant species collected from Buruli ulcer endemic and non-endemic areas in Côte d'Ivoire. (XLSX)

S2 Table Ct values obtained during RT-PCR performed on plants. (XLSX)

S3 Table Molecular identification of plant food in the feces of wild and farmed T. swinderianus grasscutters. (XLSX)

S4 Table *M*. *ulcerans* PCR results of plant species identified in the feces of wild and farmed T. swinderianus grasscutters. (XLSX)

S5 Table Comparison between *M*. *ulcerans* PCR results. (XLSX)

S1 Fig Real-time PCR graphs of plant root samples collected in Buruli ulcer endemic area of Côte d’Ivoire. (PPTX)
