# Supplementary material for: Tracing Mycobacterium ulcerans along an alimentary chain in Côte d’Ivoire: A one health perspective
Source: PLoS Negl Trop Dis. 2020 May 28;14(5):e0008228. doi: 10.1371/journal.pntd.0008228 (PMC7255608; doi:10.1371/journal.pntd.0008228)
Supplement: S1 Fig — (PPTX) [file pntd.0008228.s006.pptx]

## Slide 1
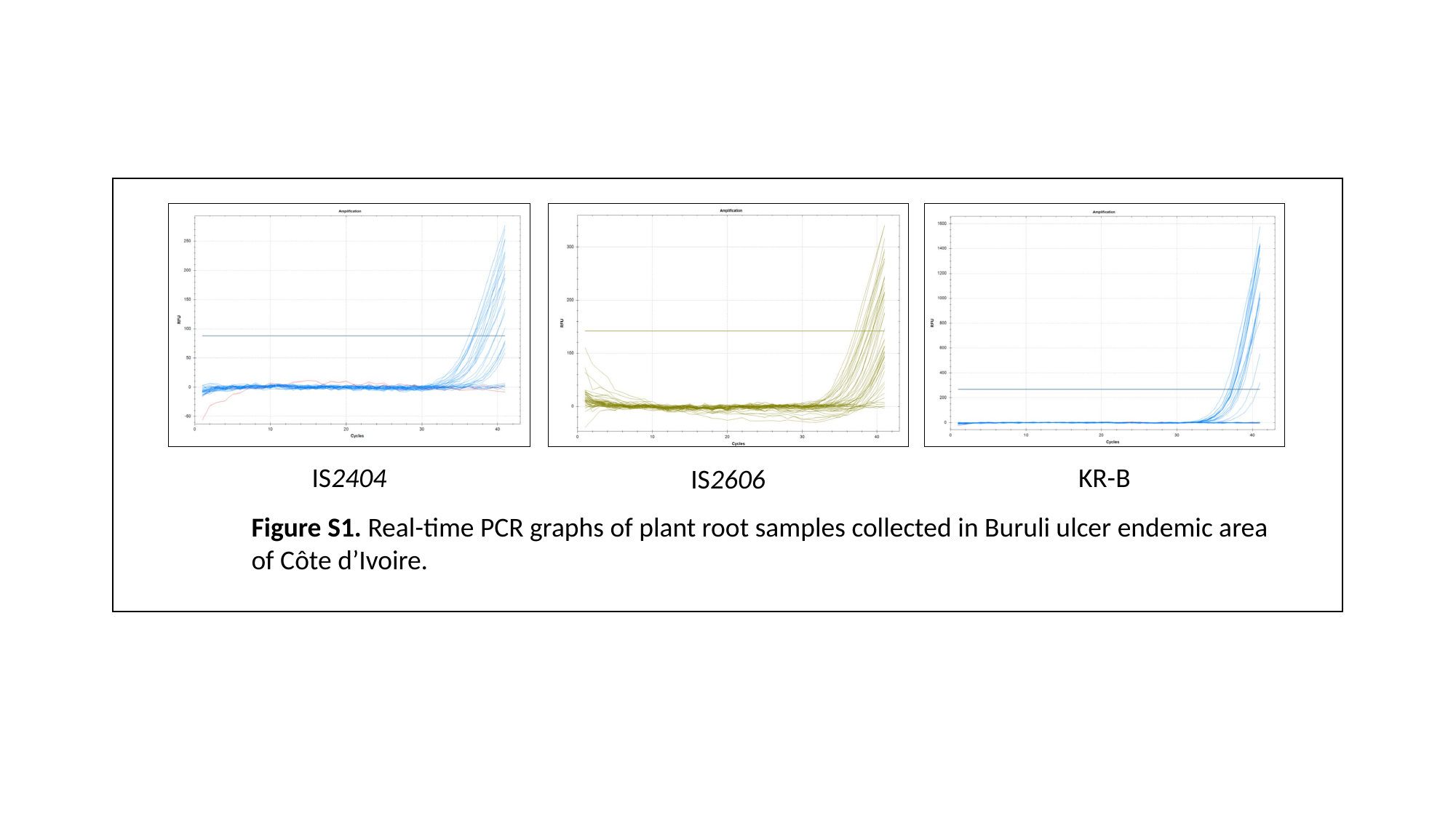

IS2404
KR-B
IS2606
Figure S1. Real-time PCR graphs of plant root samples collected in Buruli ulcer endemic area of Côte d’Ivoire.
